# A test for paedomorphism in domestic pig cranial morphology

**DOI:** 10.1098/rsbl.2017.0321

**Published:** 2017-08-09

**Authors:** Allowen Evin, Joseph Owen, Greger Larson, Mélanie Debiais-Thibaud, Thomas Cucchi, Una Strand Vidarsdottir, Keith Dobney

**Affiliations:** 1 Institut des Sciences de l'Evolution, Université de Montpellier, CNRS, IRD, EPHE, 2 Place Eugène Bataillon, 34095 Montpellier cedex 05, France; 2 Department of Archaeology, University of Aberdeen, St Mary's, Elphinstone Road, Aberdeen AB24 3UF, UK; 3 Department of Archaeology, Classics and Egyptology, University of Liverpool, 12-14 Abercromby Square, Liverpool L69 7WZ, UK; 4 Department of Archaeology, Simon Fraser University, Education Building 9635, 8888 University Dr Burnaby, Burnaby, British Columbia, Canada V5A 1S6; 5 Palaeogenomics and Bio-Archaeology Research Network, Research Laboratory for Archaeology and the History of Art, University of Oxford, Dyson Perrins Building, South Parks Road, Oxford OX1 3QY, UK; 6 UMR 7209, CNRS-Muséum National d'Histoire Naturelle, Archéozoologie, Archéobotanique: sociétés, pratiques et environnements, 55 rue Buffon, 75005 Paris, France; 7 Biomedical Center, University of Iceland, Læknagarði, Vatnsmýrarvegi 16, 101 Reykjavik, Iceland

**Keywords:** domestication, heterochrony, paedomorphism, ontogeny, *Sus scrofa*, geometric morphometrics

## Abstract

Domestic animals are often described as paedomorphic, meaning that they retain juvenile characteristics into adulthood. Through a three-dimensional landmark-based geometric morphometric analysis of cranial morphology at three growth stages, we demonstrate that wild boar (*n* = 138) and domestic pigs (*n* = 106) (*Sus scrofa*) follow distinct ontogenetic trajectories. With the exception of the size ratio between facial and neurocranial regions, paedomorphism does not appear to be the primary pattern describing the observed differences between wild and domestic pig cranial morphologies. The cranial phenotype of domestic pigs instead involves developmental innovation during domestication. This result questions the long-standing assumption that domestic animal phenotypes are paedomorphic forms of their wild counterparts.

## 1. Introduction

The process of domestication is characterized by significant changes in morphology and behaviour that differentiate domestic forms from their wild relatives [1,2]. The fact that these differences are observed consistently in a wide range of taxonomically unrelated domestic mammals implies that a similar evolutionary process is responsible for domestic phenotypes [2–5].

Traditionally, characteristics differentiating wild and domestic populations have been thought to result from changes in developmental timing (heterochrony), which lead to alterations in skeletal size and shape [6]. Many domestic animals are often described as paedomorphic (e.g. [4,7]), meaning that they retain ancestral (wild) juvenile characteristics into adulthood [8]. This paedomorphic pattern can be obtained through neoteny (also called juvenilization) characterized by a delay in shape changes relative to an unchanged size [8].

The paedomorphic hypothesis has largely been based upon studies of canids, whose novel variations in coat colour, reduced aggressiveness, and retention of social bonding and inquisitive behaviours into adulthood are traditionally cited as evidence for paedomorphism [9–11]. In addition, adult dogs possess a relative shortening of the jaw and facial region, and a widening of the palate relative to their wild ancestors [12,13]. As these changes were assumed to be the result of an allometric scaling, several studies concluded that domestic dog morphology also results from paedomorphism [12,13]. Similar arguments have been made for sheep horn form [14], and pig crania because numerous pig breeds appear to possess juvenile skull proportions (reviewed in [13]).

Despite the fact that few studies have explicitly tested the role of heterochrony and paedomorphism in shaping domestic animal diversity [9], both the lay and professional domestication literature often continues to cite the paedomorphic hypothesis as an explanation for the morphological phenotypes present in domestic animals (e.g. [15]). Two recent studies of dog cranial morphology, however, have rejected a global neotenic growth pattern for at least certain breeds (e.g. [7,16]), suggesting that paedomorphism may not explain the differences between wild and domestic populations of other taxa.

Here, in order to determine whether paedomorphism describes the distinctive cranial morphologies of domestic pigs, we contrasted the cranial shape and size of 138 West Palearctic wild boar (7 juveniles, 27 sub-adults and 104 adults) and 106 European domestic pigs (11 juveniles, 57 sub-adults and 38 adults). We initially compared the growth of wild and domestic entire cranial shape, before analysing the neurocranial and facial regions independently, because they have been identified as independent developmental modules in dogs [17]. We then quantitatively compared the growth trajectories (in terms of size, orientation and shape of trajectories) of all wild and domestic pigs before separating the early (juveniles to sub-adults) and late (sub-adults to adults) post-natal stages.

## 2. Material and methods

**Figure 1.**
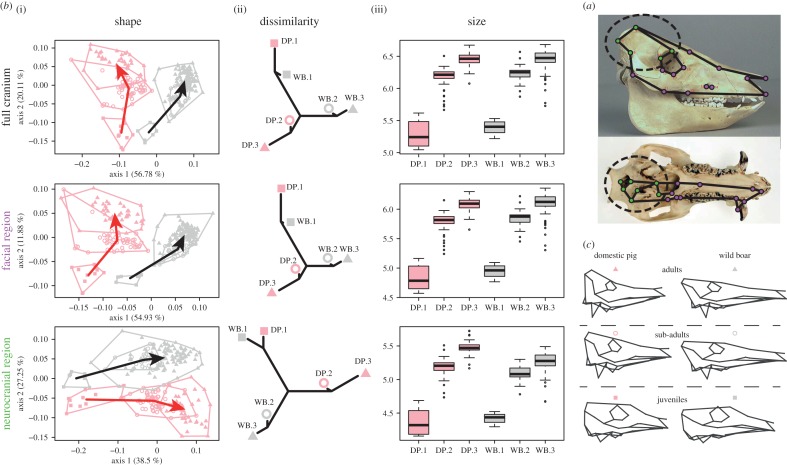
Post-natal cranial ontogeny of wild (grey) and domestic (pink) pigs. (*a*) The three-dimensional landmarks measured on the cranium divided into the facial (purple) and neurocranial (green, inside the dotted circle) regions. (*b*)(i) Cranial shape variations along the two first principal components with the ontogenetic trajectories shown as an arrow between group means pointing toward the adults. (ii) Dissimilarity in cranial shape between the groups visualized by neighbour joining networks. (iii) Growth in size (log centroid) depicted using boxplots. (*c*) Visualization of the mean shapes of each group. For the whole figure domestic pigs (DP) are represented in pink, and wild boar (WB) in grey, juveniles are numbered 1 and are represented by squares, sub-adults by number 2, and circles, and adults by number 3 and triangles.

The age class of the 244 crania analysed was assigned following Higham's protocol [18] to three age categories: juvenile, sub-adult and adult (electronic supplementary material, table S1). Thirty-six unilateral, three-dimensional coordinates (figure 1*a*; electronic supplementary material, figure S1 and table S2 [19]) were digitized from the right side of the cranium, using a Microscribe^®^ GLS (EMicroscribe Inc.). These landmarks were divided between the neurocranial and facial regions [16] (figure 1*a*). All specimen coordinates were aligned using generalized Procrustes analysis [20].

Differences in log-transformed centroid sizes and in the ratio between the sizes (log-transformed) of the facial and neurocranial regions were tested using Kruskal–Wallis tests and visualized with boxplots. Shape variation was visualized using principal component analyses (PCAs), and the differences in shape (based on PCA scores) were explored using one-way multivariate analysis of variance. Mahalanobis distances corresponding to the measure of dissimilarity between groups were derived from canonical variates analyses and visualized with neighbour joining networks. Cranial shapes of wild and domestic pigs were visualized for each of the age classes using their consensus (mean) configuration, obtained from independent superimpositions. We compared the phenotypic trajectories between the wild and domestic ontogenetic series following [21] using 1000 iterations.

Analyses were also performed on a sub-set of the original dataset, which represented two domestic breeds (Berkshire and Deutches Edelschwein) and wild boar specimens from Poland for which complete ontogenetic series were available. Where specified, *p*-values were corrected for multi-test comparisons. All analyses were carried out in R v. 3.2.1 [22], using the libraries Rmorph [23] and Geomorph [24].

## 3. Results

### (a) Morphological variation during growth

When the full cranium is analysed, the ontogenetic series of wild and domestic pigs occupy discrete positions in morphological shape space (figure 1*b*i). The two groups are clearly distinct from youth (zero to three months) to adulthood and possess increasing shape differences with age (Mahalanobis distances between juveniles *d*^2^ = 11.2, sub-adults *d*^2^ = 35.7, adults *d*^2^ = 49.4; figure 1*b*,*c*). A similar pattern is observed when the two cranial regions are analysed independently (figure 1*b*): wild and domestic pigs differ from birth (all *p* < 1 × 10^−3^), with increasing differences with age (facial region: *d*^2^ = 15.9–28.5–34.9; neurocranial region: *d*^2^ = 9.8–29.5–35.1, for juveniles, sub-adults and adults respectively).

Throughout post-natal growth, wild and domestic pigs show similar full cranium size variation (among juveniles: *χ*^2^ = 0.74, *p* = 0.39; sub-adults: *χ*^2^ = 2.3, *p* = 0.13; adults: *χ*^2^ = 0.81, *p* = 0.37; figure 1*b*iii). Similar results were obtained for the facial region (among juveniles: *χ*^2^ = 0.9, *p* = 0.34; sub-adults: *χ*^2^ = 2.73, *p* = 0.09; adults: *χ*^2^ = 2.9, *p* = 0.09; figure 1). The neurocranial region does not differ in size between wild and domestic juveniles (*χ*^2^ = 0.1, *p* = 0.75). Domestic sub-adults and adults, however, possess a larger neurocranial region than their wild relatives (among sub-adults: *χ*^2^ = 11.4, *p* = 0.0007; adults: *χ*^2^ = 57.39, *p* = 3.5 × 10^−14^; figure 1*b*iii).

**Figure 2.**
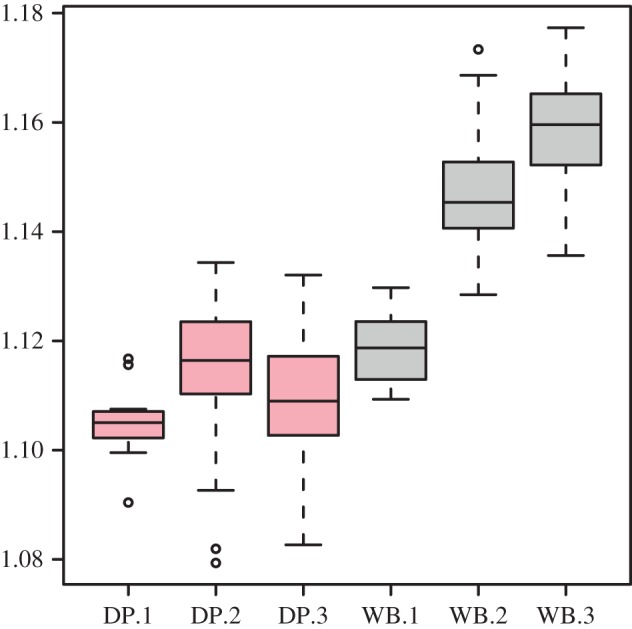
Evolution through growth of the size ratio between the facial and neurocranial regions. (Online version in colour.)

As a consequence, the size ratio between the facial and neurocranial regions changes in a different manner in wild and domestic pigs during ontogeny (figure 2). While wild boar display an increase in the ratio throughout growth (all *p* < 0.05), domestic pigs show only an increase between the juvenile and sub-adult stages (*χ*^2^ = 11.83, *p* = 0.0006) followed by a decrease between the sub-adult and adult stages (*χ*^2^ = 7.6, *p* = 0.006; figure 2). This pattern is responsible for the larger neurocranial region observed in domestic pigs, while the size of the facial region is identical in wild and domestic pigs in these two age classes (figure 1*b*iii).

### (b) Growth trajectories

In analyses of the entire skull and the separate regions, the ontogenetic trajectories for wild and domestic pigs differ in both shape and orientation, but not in length (table 1 and figure 1*b*i). However, the observed amount of change between the sub-adults and adults is significantly greater in domestic pigs than in wild boar for all structures (table 1).

**Table 1.** Ontogenetic trajectories. Differences in trajectory length, shape and orientation. *p*-values in italics remain significant after correction for multi-test comparisons. Δ*d*: absolute differences between path distances, cor.: principal vector correlation, Δshape: shape differences.

	all specimens						juveniles/sub-adults				sub-adults/adults			
	Δ*d*	*p*-value length	cor.	*p*-value orientation	Δshape	*p*-value shape	Δ*d*	*p*-value length	cor.	*p*-value orientation	Δ*d*	*p*-value length	cor.	*p*-value orientation
cranium	0.023	0.086	*0*.*756*	*0*.*005*	*0*.*138*	*0*.*010*	0.007	0.623	*0*.*834*	*0*.*001*	*0*.*030*	*0*.*001*	*0*.*606*	*0*.*001*
facial region	0.01	0.439	*0*.*830*	*0*.*001*	*0*.*163*	*0*.*001*	0.014	0.293	*0*.*892*	*0*.*005*	*0*.*024*	*0*.*002*	*0*.*640*	*0*.*001*
neurocranial region	0.03	0.115	*0*.*920*	*0*.*005*	*0*.*148*	*0*.*010*	0.009	0.607	*0*.*923*	*0*.*001*	*0*.*041*	*0*.*003*	*0*.*741*	*0*.*001*

These results include all available specimens and are largely congruent with analyses restricted to the two domestic breeds (Berkshire and Deutches Edelschwein) and single wild population (Poland), where complete ontogenetic series were available (electronic supplementary material, figure S2).

## 4. Discussion

At no point during development does the cranium of a domestic pig resemble that of a juvenile wild boar, a prerequisite for the paedomorphic model [25]. Moreover, significant differences in cranial shape are already present in wild and domestic pigs at the juvenile stage, which indicates that the differences in adult morphology are at least partially established during prenatal growth. Thus, the ontogenetic mechanisms responsible for the observed differences are initiated before birth.

Wild and domestic pigs undergo similar amounts of change in cranial morphology during post-natal development, but they follow different ontogenetic paths that further reinforce the juvenile cranial shape differences. Therefore, adult domestic pig cranial morphology is not the result of a truncated ancestral ontogenetic trajectory, as assumed by the paedomorphic model. Thus, in contradiction to an extensive body of literature on the domestication process (e.g. [12,13], with a notable exception [16]), we can, therefore, reject the hypothesis that the domestic pig cranium is paedomorphic.

However, the early cessation of the increase in the face/neurocranium size ratio observed in domestic pigs may appear congruent with a paedomorphic pattern. The ‘domestication syndrome’ in mammals includes a shortening of the face [7], which in domestic pigs appears to be the result of both a change in facial shape (which becomes shorter and wider) and an increase in neurocranial size.

The differences between wild and domestic pig ontogenetic trajectories are much greater than those previously documented for dogs [16]. Pig and wild boar crania also show more pronounced differences in adult shape, compared with the dog/wolf results [16]. Analysing a greater number of wild and domestic pairs will establish whether these ontogenetic patterns are generalizable in other taxa.

Domestication is a long, complex, continuous and on-going process which, for pigs, began some 10 500 years ago [26]. Unfortunately, the scarcity of complete pig crania in the archaeological record restricts the potential to explore the initial phases of domestication and determining the temporal emergence of these developmental alterations. The process of domestication also induced other morphological changes, including a greater rate of asymmetry in domestic forms [27,28] that may have resulted from environmental or genetic stress [29] and likely also develop during growth, all of which deserve to be explored in further studies.

## 5. Conclusion

Domestic pigs are not simply paedomorphic wild boar. Developmental changes initiated before birth and accentuated by distinct post-natal growth trajectories are responsible for the domestic pig's cranial morphology. This paper highlights the importance of development in understanding domestic morphologies and the diversity of the resulting patterns (e.g. dogs versus pigs). Our results do not preclude the possibility that paedomorphism may exist in other traits or in other species, but claims for such require rigorous testing. Because wild and domestic pigs differ at the earliest developmental stages, additional studies of embryogenesis are needed to better understand the evolution of domestic phenotypes.

## Supplementary Material

SI Figure 1: A depiction of the location and number of homologous landmarks used in the analysis.

## Supplementary Material

SI Figure 2: Results of analyses performed on the three ontogenetic series for which specimens in the three classes of age were available.

## Supplementary Material

SI Table 1: List of all specimens included in the study. Juvenile: 0-3 months, before the eruption of all deciduous teeth, sub-adult: 3-15 months, and adult: over 15months, after deciduous teeth being replaced by permanent teeth.

## Supplementary Material

SI Table 2: Description of landmarks used, and of the sub-regions of the crania.

## Data accessibility

The datasets supporting the results of this article are available in the Dryad Digital Repository (http://dx.doi.org/10.5061/dryad.c3f25) [19].
